# Performance evaluation of a combination *Plasmodium* dual-antigen CRP rapid diagnostic test in Lambaréné, Gabon

**DOI:** 10.1007/s15010-024-02366-y

**Published:** 2024-08-23

**Authors:** Ayodele Alabi, Fungai P. Musangomunei, Fabrice Lotola-Mougeni, Juste C. Bie-Ondo, Kristin Murphy, Paulin N. Essone, Anita L. Kabwende, Saidou Mahmoudou, Aurélien Macé, Victoria Harris, Michael Ramharter, Martin P. Grobusch, Maria Yazdanbakhsh, B. Leticia Fernandez-Carballo, Camille Escadafal, Peter G. Kremsner, Sabine Dittrich, Selidji T. Agnandji

**Affiliations:** 1 Biomedicine and Social Sciences research group, Department of Biologicals and Therapeutics, Centre de Recherches Médicales de Lambaréné, CERMEL, BP 242, Lambaréné, Gabon; 2 Institut für Tropenmedizin, Universitätsklinikum Tübingen and German Centre for Infectious Diseases Research (DZIF), Tübingen, Germany; 3 Leiden University Center for Infectious Diseases (LU-CID), Leiden University Medical Center, Leiden, The Netherlands; 4 London School of Hygiene and Tropical Medicine, London, UK; 5 FIND, Geneva, Switzerland; 6 Department of Tropical Medicine, Department of Internal Medicine I, Bernhard Nocht Institute for Tropical Medicine, University Medical Centre Hamburg-Eppendorf, Hamburg, Germany; 7 Center of Tropical Medicine and Travel Medicine, Department of Infectious Diseases, Amsterdam Public Health, Amsterdam University Medical Centres, location Amsterdam, Amsterdam Infection & Immunity, University of Amsterdam, Amsterdam, The Netherlands; 8 Nuffield Department of Medicine, University of Oxford, Oxford, UK; 9 Deggendorf Institut of Technology, European Campus Rottal-Inn, Deggendorf, Germany; 10 Institute of Medical Microbiology, University Hospital Münster, 48149, Domagkstraße 10, Münster, Germany

**Keywords:** Acute febrile illness, Malaria, Antimicrobial stewardship, C-reactive protein, Gabon

## Abstract

**Purpose:**

The consequent use of malaria rapid diagnostic tests (RDTs) preceding a treatment decision has improved the global management of malaria. A combination RDT, including an inflammation marker to potentially guide antibiotic prescription, could improve the management of acute febrile illness (AFI).

**Methods:**

We performed a prospective, cross-sectional study in Gabon evaluating the STANDARD Malaria/CRP DUO (S-DUO) RDT. Participants aged 2 to 17 years with fever at presentation and/or a history of fever < 7 days were enrolled. Expert microscopy, SD Bioline Malaria Ag P.f/Pan test for malaria detection, and NycoCard CRP device for CRP were used as comparators. AFI cases were classified on a spectrum encompassing bacterial vs. non-bacterial infection.

**Results:**

415 participants with AFI were enrolled. S-DUO RDT sensitivity and specificity for malaria detection vs. microscopy were 99·1% (95·2–100%) and 72·7% (64·3–80·1%); and for CRP detection (20 mg/L and above) 86·9% (80–92%) and 87% (79·2–92·7%), respectively. The difference in CRP levels between bacterial infection (mean = 41·2 mg/L) and other causes of fever, measured from our study population using the Nycocard device, was statistically significant (*p* < 0·01); CRP precision-recall AUC to distinguish bacterial infection class vs. non-bacterial classifications was 0·79.

**Conclusion:**

S-DUO RDT is suitable for malaria detection in moderate-to-high malaria transmission settings such as in Lambaréné; however, a CRP band detection limit > 40 mg/L is more adequate for indication of antibiotic prescription for AFI cases in Gabon.

## Introduction

The increased availability of malaria rapid diagnostic tests (RDTs) has improved clinicians’ abilities to diagnose and treat malaria, especially in malaria-endemic regions. Their appropriate use, in combination with clinical assessments, allows for stewardship of highly effective antimalarial drugs such as artemisinin-based combination treatments (ACTs) and helps mitigate the development of resistance against these drugs.

A negative malaria RDT creates a diagnostic uncertainty where fever determined as non-malarial in aetiology cannot be reliably differentiated as either bacterial or non-bacterial, notwithstanding the question of whether a malaria case might be complicated by a bacterial co-infection such as a nontyphoidal salmonella infection. As a result, the practice of presumptive antibiotic administration continues to flourish. This misuse of antibiotics permits the emergence of drug-resistant pathogens. It adds to the growing burden of antimicrobial resistance, which has been declared a top-ten global health threat. [1]

C-reactive protein (CRP), a human pro-inflammatory marker, has been investigated as a biomarker to differentiate bacterial from non-bacterial infection in febrile participants, [2, 3] with the combined use of rapid tests for CRP and malaria being recommended for consideration in febrile case management. It has previously been shown that CRP-guided therapy can reduce inappropriate antibiotic prescriptions and improve clinical outcomes in sub-Saharan Africa. [4] This study describes the evaluation of a first-of-its-kind combination (dual antigen and CRP) RDT, the STANDARD™ Q Malaria/CRP DUO (S-DUO; SD Biosensor, Deogyeong-daero, South Korea) test, for the diagnosis of malaria (malaria antigen band); in distinguishing bacterial infection and as a guidance for the prescription of antibiotics (CRP band to detect above 20 mg/L); in a clinical setting in Lambaréné, Gabon, a highly malaria-endemic region.

## Methods

### Study design and population

This study was an ancillary investigation into the performance of a dual RDT, nested into the Biomarker for Fever-Diagnostic study (BFF-Dx), which was a prospective, multicentre, cross-sectional healthcare-based study in Brazil, Gabon, and Malawi that looked to evaluate host biomarkers of fever to support the development of point-of-care diagnostic tests to guide antibiotic use in cases of acute febrile illness (AFI) [5].

The study was conducted at the Centre de Recherches Médicales de Lambaréné (CERMEL) in Lambaréné, Gabon [6]. Lambaréné is located in the province of Moyen-Ogooué, with a population estimated at 38,775 inhabitants according to the last nationwide census conducted in 2013 [7]. Lambaréné is 254 km by road from Libreville, the capital of Gabon and crossed by the Ogooué River.

Children aged 2 to 17 years presenting to the CERMEL clinic with acute febrile illnesses (temperature of more than 38 °C oral or tympanic temperature, or more than 37.5 °C axillary or forehead/skin temperature at initial evaluation, or with history of fever in less than seven days of symptoms at enrolment) were recruited for the study. Enrolment was also based on participants’ willingness to have a study follow-up visit if required (approximately two weeks after the enrolment visit) and to have HIV testing performed. After the screening, individuals clinically assessed to be in a critical condition or presenting with signs of critical illness as defined by WHO guidelines [8] (for children: extensive vomiting, active seizure or recent history of seizures, altered mentation, inability to feed) were excluded.

### Rapid diagnostic tests

The index test being evaluated, the S-DUO Test, is a RDT that contains two connected test cassettes, one for malaria and one for CRP. The device is a lateral flow immunochromatographic assay that detects histidine-rich protein 2 (HRP-2) antigen-specific for *P. falciparum* and pLDH specific for *Plasmodium* species. In parallel to the S-DUO test, another malaria RDT (SD Bioline Malaria Ag P.f/Pan test (Abbott, Illinois, US), also HRP-2/pLDH-based) that is widely available and used routinely in health care in Gabon was performed to allow clinicians to make treatment decisions for patients.

Upon presentation to the CERMEL clinic for evaluation of fever, all enrolled participants were screened for malaria using the SD-Bioline and S-DUO RDTs following the manufacturer’s instructions. Study nurses were trained on theoretical and hands-on performance and reading of the S-DUO Test. The malaria RDTs were read and interpreted in a blinded manner, with two separate nurses assigned to read the RDTs: one to interpret the SD-Bioline RDT and another to the S-DUO Test. The malaria RDTs were considered negative when only the control line was visible, positive when both control and test lines were visible, and invalid when a control line was not seen, irrespective of whether a test line was visible. Test lines were documented as either faint or deep and were considered present in either case. Concerning the S-DUO Test, the CRP cartridge displays a visible band at the CRP area of the test device if the sample has a level at or above the detection limit of 20 milligrams per litre (mg/L). A visible band at the control area was required to validate the test. For this analysis, a visible control band and a band at the CRP area of the device constituted a positive CRP index test.

### Comparator tests

Malaria RDT results were compared with expert microscopy. All enrollees had two blood smears prepared on microscope slides from the same finger prick used for RDTs. Air-dried blood smears were stained with fresh Giemsa stain using standard procedure [9]. All blood smears were double-read by two experienced malaria microscopists blinded to each other’s results and the results of the RDTs. The second slide was stored for archiving purposes. Diagnosis of malaria was based on identifying asexual stages of *Plasmodium* spp. on thick blood smears. Parasite density was determined by counting the number of asexual parasites against approximately 200 leukocytes on the thick blood film and converted to parasites per microliter using an assumed total white blood cell count of 8000 cells/µL. [9]

For the measurement of CRP, the reference test was performed using the NycoCard CRP device (Abbott, Illinois, US), which gives a quantitative result with a measurement range of five to 120 mg/L. The NycoCard is a solid phase, sandwich-format, immunometric assay. Samples with NycoCard CRP levels > 20 mg/L constituted a positive CRP reference test; this cut-off value reflected the detection limit of the S-DUO test, allowing for adequate comparisons between both tests. A CRP test threshold of 20 mg/L is estimated to have a sensitivity of above 85% when used to detect bacterial infection, particularly in studies in Southeast Asia. [10]

### Clinical studies

Onsite microbiology investigations were performed to identify the cause of fever (bacterial or non-bacterial) in line with the presenting symptoms. [10] Staff underwent training and reviewed detailed sample handling and processing standard operating procedures before recruitment. On enrolment, clinicians recorded the history of present illness, past medical history, and a physical examination on paper case-report forms. RDTs were performed, and slides for microscopy were prepared from finger-prick blood samples. Venous blood (≥ 12 ml) was collected aseptically for hemocytometry, blood culture, serology, and plasma PCR. A mid-stream urine sample from each participant was collected in sterile tubes and stored.

We employed a two-step process, in which the initial classification of bacterial vs. non-bacterial causes of fever by microbiological testing, was followed by a retrospective review of a summary case file that included the patient’s history, clinical data, and laboratory findings by a panel of three independent expert clinicians for cases that remained unclassified by the former. [5, 11] Classification during the first step was based on the results of a complete clinical workup, such as with sample cultures, RDTs, ELISAs, PCRs, etc. (Table 1). In this first step, cases that met bacterial and non-bacterial infection criteria were classified as bacterial infections. After review, each case was classified into a final category: one of the five overarching categories: bacterial infection (BI), non-bacterial infection (NB), ‘probable bacterial infection’ (PB), ‘probable non-bacterial infection’ (PNB), or indeterminate cause of fever (Ind); the ‘probable’ categories encompassed cases in which two of the three experts agreed on the cause of AFI, either bacterial or non-bacterial; the ‘indeterminate’ category encompassed cases in which none of the experts could reach an agreement. Associations between CRP band results and final classifications were assessed to evaluate the clinical relevance of the S-DUO CRP band.

**Table 1:** Criteria for first step of cases classification between bacterial and non-bacterial infection. Adapted from Escadafal et al. [5]

Diagnostic method	Bacterial infection	Non-bacterial infection
Microscopy/culture	Bacteria	Parasite/Fungus
Serology (ELISA/RDT)	*Leptospira* spp. *Streptococcus pyogenes* *Streptococcus pneumoniae*	Ddngue Zika chikungunya rotavirus adenovirus *Cryptococcus s*pp.
Multiplex PCR (Respiratory swabs)	Respiratory bacteria	Respiratory viruses

**Table 2:** Baseline clinical characteristics of study participants according to analysis grouping

Variable	Intention-to-test population	Per Protocol population	Excluded from per protocol population	*p*-value
Number of participants	415	245	170	-
Age, years (SD)	7·2 (4·2)	7·1 (4·0)	7·4 (4·4)	0·5354
Female sex	228 (54·9%)	140 (57·1%)	88 (51·8%)	0·279
Illness duration, days (SD)	3·1 (1·5)	3·1 (1·5)	3·0 (1·5)	0·3044
Malaria infection^*#^	171 (41·9%)	113 (46·1%)	58 (35·6%)	0·035
Parasite density (per µL of blood)^+^				0·229
1 to < 500	35 (8·8%)	24 (10·1%)	11 (6·9%)	-
500 to < 5000	45 (11·3%)	30 (12·6%)	15 (9·4%)	-
*≥* 5000	81 (20·4%)	52 (21·9%)	29 (18·1%)	-
CRP level, mg/L (SD)*	43·4 (41·0)	42·8 (41·1)	44·2 (40·9)	0·391
HIV infection	5 (1·2%)	5 (2·0%)	0 (0%)	0·082

SD = Standard deviation

- Not applicable

*According to comparator tests

#For malaria infection, *N* = 408 for ITT, *N* = 163 for excluded from PP, due to missing malaria microscopy results

+For parasite density, *N* = 398 for ITT, *N* = 238 for PP population, *N* = 160 for excluded from PP due to missing parasite density calculations

### Data management and analysis

Investigators used handwritten case report forms for data capture, which were then entered into a secure database. Data was transferred to R software version 4·2·0 (Year 2022) (www.r-project.org) for analysis. Descriptive statistics were presented as means, ranges, and interquartile ranges (IQRs) for continuous variables; categorical variables, including demographic characteristics and RDT band results, were summarised using frequency and percentage. Two-sided P values < 0.05 were considered statistically significant. Participants with invalid or missing index or reference results were excluded from the per-protocol (PP) population and subsequent analysis. For malaria microscopy, a result of ‘positive’ or ‘negative’ was required for inclusion, though speciation and parasite density was also reported for most positive samples.

To compare the mean CRP in the final classification groups and predicted categories versus the actual categories for agreement, simple linear regression and McNemar’s Chi-squared test were used, respectively. The random forest model with leave-one-out cross-validation was used to evaluate if a final classification can be predicted using the CRP level. Due to imbalanced data, we used the precision-recall area under the ROC curve (PRAUC) to compare the model’s performance [12]. Different models were used in redefining the final classification: (1) strict classification i.e., bacterial infection (excluding probable bacterial) vs. non-bacterial infection (excluding probable non-bacterial); (2) loose classifications i.e., bacterial infection (excluding probable bacterial) vs. probable non-bacterial infection, bacterial infection (excluding probable bacterial) vs. non-bacterial infection (including probable non-bacterial), and bacterial infection (including probable bacterial) vs. non-bacterial (including probable non-bacterial); and (3)PRAUC.

### Ethical approval

Ethical approval was obtained from the Institutional Ethics Committee of CERMEL and the Gabonese National Ethics Committee. Individual informed consent was obtained in accordance with the ethical standards set by the committees. Written informed consent was obtained from adults and parents/guardians of adolescents/children and written assent from minors aged 13–17 years before study-related procedures.

### Role of funders

The funders had no role in data collection, analysis, interpretation of the results, or decision to publish.

## Results

**Fig. 1:**
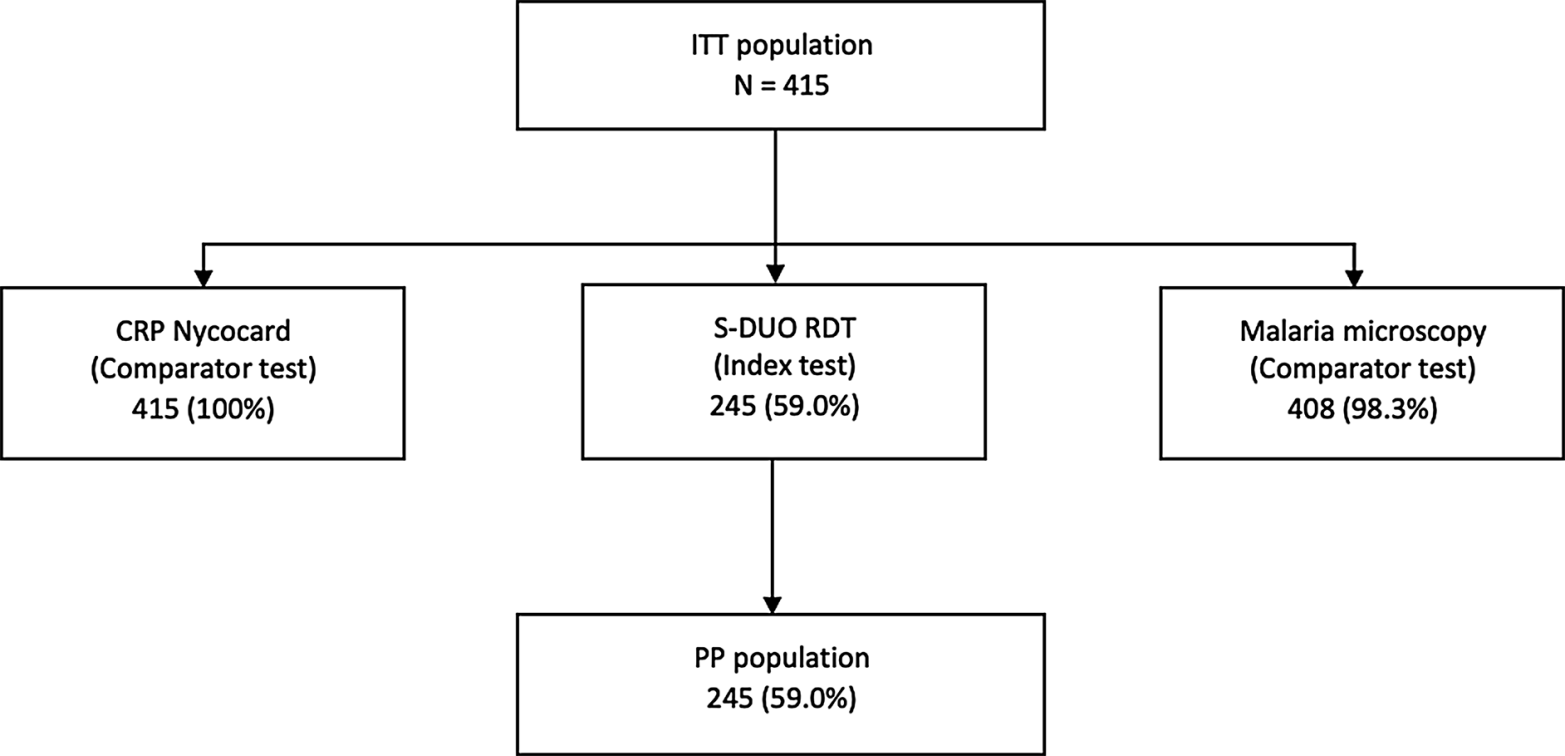
Study flow diagram, showing participants for whom complete test results were available for the different testing methods. ITT = intention-to-test; PP = per protocol

From May through November 2019, 415 children were enrolled in the study (Fig. 1). The mean age of enrollees was seven years, and the majority were female (55%). Fever occurrence at presentation was observed in 21% (87/415) of participants, and the onset of symptoms was, on average, three days before presentation.

Malaria microscopy results were available for 408 (98·3%) participants, of which 245 (59·0%) had paired S-DUO RDT results, making up the Per Protocol (PP) analysis population. Participants excluded from the PP analysis did not differ in age, gender, parasite density, or mean CRP levels (mg/L) in serum. Malaria was detected by microscopy in 42% (171/408) of participants, 92% (157/171) of which were *Plasmodium falciparum* (*P.f*) mono-infection. *Plasmodium malariae* (one co-infection with *P.f*) and *Plasmodium ovale* (co-infection with *P.f*) were identified in 5% (8/171) and 0.6% (1/171), respectively; the geometric mean parasite density was 3867 parasites/ul (range: 54-211014). Details of other patient characteristics categorised according to the analysis group, are shown in Table 2. When applying the pre-defined AFI classification criteria, 58% (240/415) of cases were classified as non-bacterial, 17% (70/415) as probable non-bacterial, 12% (50/415) as indeterminate, 10% (41/415) as bacterial, and 3% (14/415) as probable bacterial infection by the experts (submitted).

**Table 3:** Diagnostic performance of STANDARD Q Malaria/CRP DUO RDT vs. microscopy, SD-Bioline, and NycoCard CRP device

STANDARD Q Malaria/CRP DUO RDT	Microscopy as comparator test			
	Sensitivity (95% CI)	Specificity (95% CI)	PPV (95% CI)	NPV (95% CI)
**HRP-2 or pLDH band**	99·1% (95·2–100%)	72·7% (64·3–80·1%)	75·7% (67·9–82·3%)	99·0% (94·4–100%)
**HRP-2 band**	98·2% (93·8–99·8%)	74·2% (65·9–81·5%)	76·7% (68·8–83·2%)	98% (93–99·8%)
**pLDH band**	72·6% (63·4–80·5%)	97·0% (92·4–99·2%)	95·3% (88·5–98·7%)	80·5% (73·5–86·4%)
**STANDARD Q Malaria/CRP DUO RDT**		**SD-Bioline as comparator test**		
**Positive band**	**Per protocol population**	**Agreement**	Kappa (SE*)	**p-value**
HRP-2 or pLDH	148 (60·4%)	87·4%	0·81 (0·04)	< 0·001
HRP-2 and pLDH	83 (33·9%)			
HRP-2 only	62 (25·3%)			
pLDH only	3 (1·2%)			
**STANDARD Q Malaria/CRP DUO RDT**	**NycoCard CRP device as comparator test**			
	**Sensitivity (95% CI)** 86·9% (80–92%)		**Specificity (95% CI)** 87·0% (79·2–92·7%)	
**CRP band**				

*Standard error

HRP2 = Histidine Rich Protein 2; pLDH = Plasmodium Lactate Dehydrogenase; PPV = Positive Predictive Value; NPV = Negative Predictive Value

### The STANDARD Q Malaria/CRP DUO RDT shows good accuracy in diagnosing malaria

The S-DUO RDT was highly accurate in diagnosing malaria when the infection was present, and was in good agreement with SD-Bioline RDT readings. Using microscopy as a comparator, the sensitivity and specificity of the S-DUO RDT were 99·1% (95·2–100%) and 72·7% (64·3–80·1%), while the positive and negative predictive values (PPV and NPV) were 75.7% (67·9–82·3%) and 99·0% (94·4–100%), respectively (Table 3). When stratified by malaria antigen detection band, sensitivity and specificity were 98·2% (93·8–99·8%) and 74·2% (65·9–81·5%) for the HRP-2 band, and 72·6% (63·4–80·5%) and 97% (92·4–99·2%) for the pLDH band, respectively. There was good agreement between the S-DUO and SD-Bioline RDTs with a kappa value of 0·81.

**Table 4:** Random Forest model sensitivity, specificity, and kappa for discordance

Model	Accuracy [95% CI]	Kappa	Sensitivity [95% CI]	Specificity [95% CI]
Bacterial vs. probable non-bacterial	83 [74–89]	63	75 [59–87]	87 [77–94]
Bacterial vs. non-bacterial	89 [84–92]	40	30 [17–47]	99 [97–100]
Bacterial vs. probable non-bacterial and non-bacterial	91 [87–94]	40	30 [17–47]	99 [97–100]
Bacterial and probable bacterial vs. probable non-bacterial and non-bacterial	87 [83–91]	42	39 [26–53]	96 [93–98]

HRP2 = Histidine Rich Protein 2; pLDH = Plasmodium Lactate Dehydrogenase; PPV = Positive Predictive Value; NPV = Negative Predictive Value.

The malaria positivity rates were 46·1% (113/245), 58·0% (142/245), and 60·4% (148/245) using microscopy, SD-Bioline, and S-DUO, respectively. HRP-2 and pLDH positivity were 58·0% (142/245) and 24·5% (60/245) for SD-Bioline, and 59·2% (145/245) and 35·1% (86/245) for S-DUO, respectively. The positivity rate was higher in males than in females for both the HRP-2 and the pLDH components of the RDTs. There was no statistically significant association for gender and parasite density.

### The CRP detection limit of 20 mg/L does not distinguish bacterial from non-bacterial AFI

Using the NycoCard CRP device as a comparator, the sensitivity and specificity of the S-DUO RDT CRP detection band were 86·9% (95% CI: 80–92%) and 87% (95% CI: 79·2–92·7%), respectively.

In the PP population, 133/245 (54·3%) were CRP band-positive, and 112/245 (45·7%) were CRP band-negative. Of the cases in which the CRP band was positive, 46·6% (62/133), 17·3% (23/133), 15·8% (21/133), 15·8% (21/133) and 4·51% (6/133) were classified as non-bacterial infection, probable non-bacterial infection, bacterial infection, indeterminate, and probable bacterial infection, respectively. Of the cases in which the CRP band was negative, 70% (75/112), 14·3% (16/112), 11·6% (13/112), 4·46% (5/112), and 2·68% (3/112) were classified as NB, PNB, Ind, BI, and PB, respectively.

### Study population-derived CRP values and aetiology of AFI

**Fig. 2:**
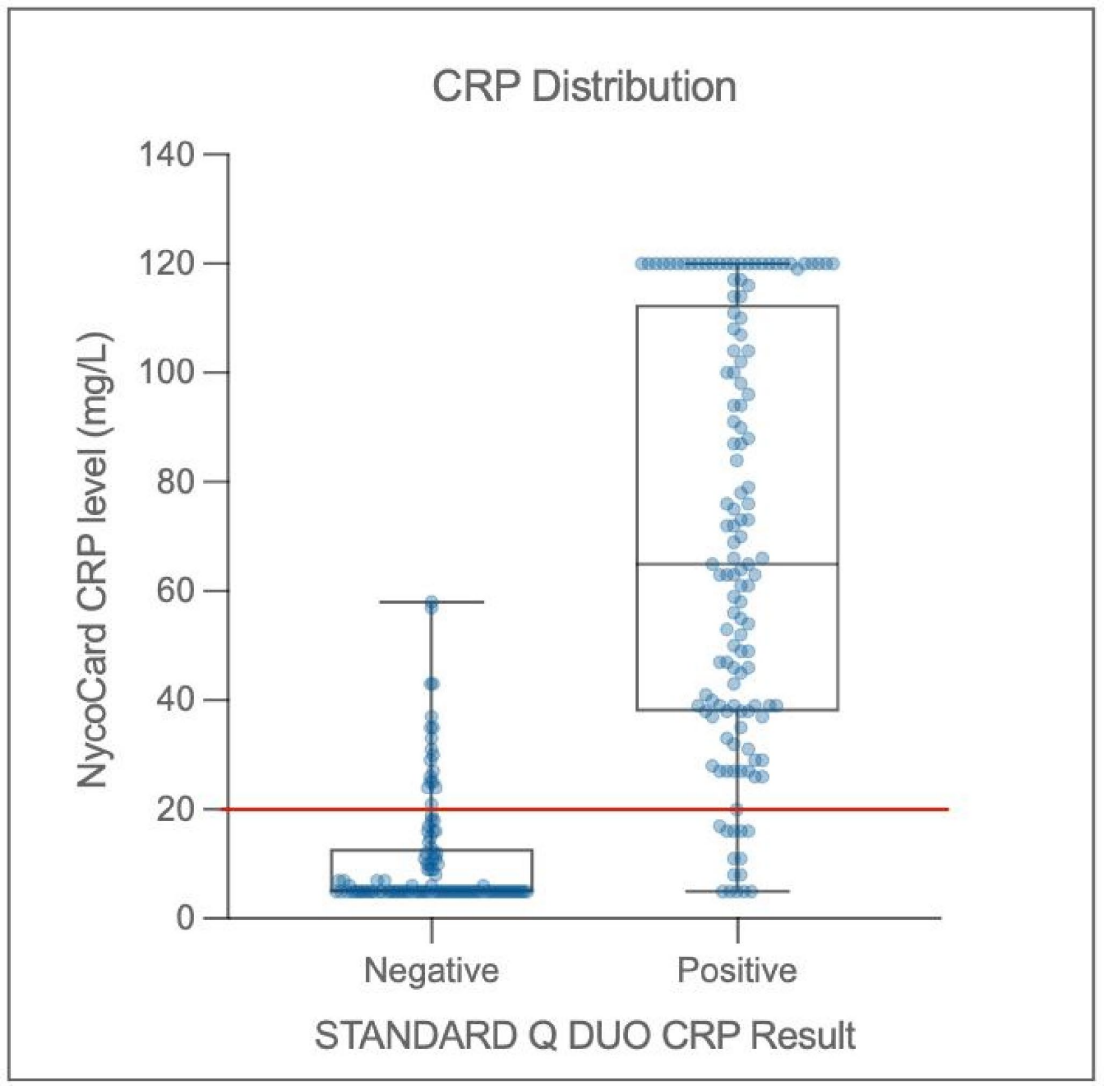
CRP distribution by Nycocard device vs. S-DUO RDT result in the Per Protocol population. Median values and interquartile ranges shown in box plots, the dots represent individual samples. The red line indicates detection limit of S-DUO RDT (20 mg/L)

The median CRP level in the study population was 27 mg/L, as measured by the Nycocard device. The presence of malaria infection was associated with a 27·6 mg/L increase in CRP levels (95% CI 20·0–35·3 mg/L; p = < 0.001). For S-DUO RDT negative and positive samples, the median (IQR) NycoCard CRP level was 5 mg/L (5–12.5) and 65 mg/L (38–111), respectively (Fig. 2). Most false positive and false negative samples, as identified by S-DUO RDT, had CRP concentrations that were very close to the detection limit of 20 mg/L.

**Fig. 3:**
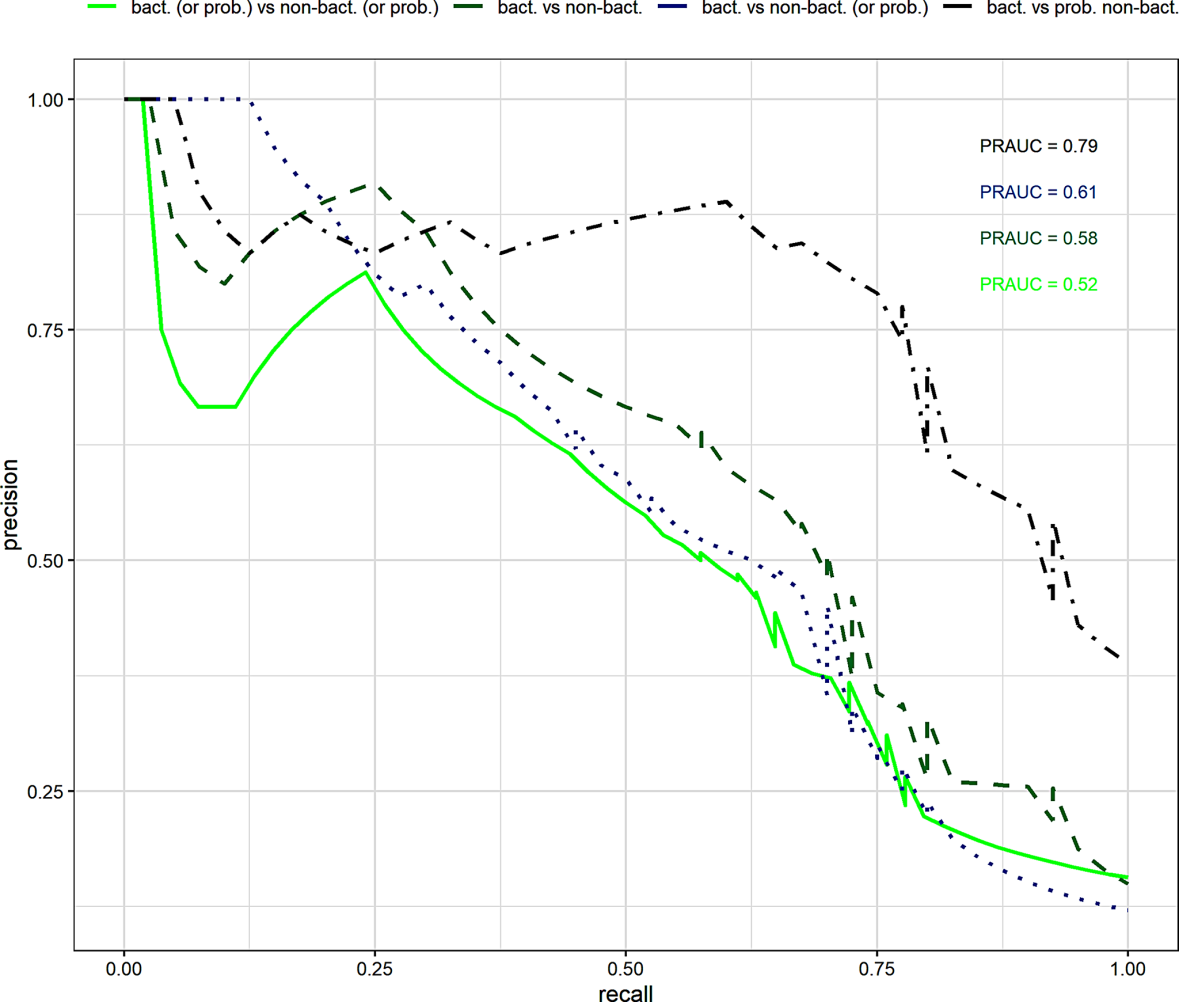
PRAUC using CRP for classification with random forest model; bacterial (including probable bacterial) vs. non-bacterial (including probable non-bacterial) denoted in light green, bacterial vs. non-bacterial (excluding probable non-bacterial) denoted in dark green, bacterial vs. non-bacterial (including probable non-bacterial) denoted in blue, bacterial vs. probable non-bacterial denoted in black, respectively. A high PRAUC represents both high precision (in this case, a low false positive rate) and high recall (a low false negative rate)

The mean (SD) CRP level for bacterial infection, probable bacterial infection, probable non-bacterial infection, non-bacterial infection, and indeterminate were 41·2 mg/L (40), 23·3 mg/L (38·1), 22·1 mg/L (43·3), 21·0 mg/L (39·1), and 31·1 mg/L (44·5), respectively. There was a significant difference in CRP levels between bacterial and non-bacterial infection classifications (*p* = 0·008). Using the random Forest model, CRP level also showed a sensitivity and specificity of 30% (17–47) and 99% (97–100), respectively; the kappa coefficient revealed moderate agreement between the prediction and actual classification (Table 4). PRAUC values for loose classifications, bacterial vs. probable non-bacterial, bacterial vs. non-bacterial (including probable non-bacterial), bacterial (including probable bacterial) vs. non-bacterial (including probable non-bacterial) and strict classification, bacterial vs. non-bacterial (excluding probable) classes were 0·79, 0·61, 0.52, and 0·58, respectively (Fig. 3).

## Discussion

The scaling-up of the use of malaria RDTs has been a success story; [13] however, there continues to be a need for innovation and adaptation of these point-of-care (POC) tests. Predicaments include the threat of a decrease in sensitivity of first-generation malaria RDTs, [14] inappropriate use of RDTs by health care workers, [15, 16] the prozone effect, [17] epidemiological changes due to evolutionary pressures as seen with HRP-2 deletions, [18] and continued overuse of antibiotics. This study determined the performance of the S-DUO RDT in a clinical setting in semi-rural Lambaréné, Gabon.

The S-DUO RDT produced a high sensitivity and modest specificity for detecting malaria using the HRP-2 or pLDH antigens compared to microscopy. The lack of false negative results means S-DUO RDT was suitable for capturing all study participants that required antimalarials and the relative absence of the prozone effect in our cohort; these results are to be expected from a combination (HRP2 + pLDH) malaria RDT. The high percent agreement (87.4%) and kappa statistic (0.81), which are measures of intra-measurement reliability, between SD Bioline and S-DUO RDT, verify that the S-DUO RDT has performed well for diagnosing malaria when compared to the standard of care in this setting.

The modest specificity might be due to stable malaria transmission and prolonged clearance of HRP-2 from the blood, [19] resulting in false positive (FP) results from prior infection. RDT is more sensitive than microscopy, and this is also a plausible reason in our cohort, which included participants with a history of fever occurring within 7 days before enrollment [20]. We noted elevated FP rates in dengue (53%, 9/17) and chikungunya (33%, 5/15) cases that underwent S-DUO RDT testing in our cohort. FP rates for Zika were relatively low (5%, 2/14), meaning the presence of these arboviruses had an impact on performance of the device. SD Bioline RDT FP rates for dengue, chikungunya, and Zika cases were 38% (13/24), 66% (10/15), and 43% (6/14), respectively. These results might be linked to the manufacturing process or the antibody used in the RDT devices.[21] There were no similar observations for other cases. A high NPV and modest PPV confirm that the S-DUO RDT is suitable for malaria detection in moderate-to-high malaria transmission settings, although unlikely to impact antimalarial overmedication. In cases of fever with a positive malaria RDT, a search for an alternate cause of fever or co-infection should still be encouraged, in arboviral endemic regions clinicians should consider these infectious agents or cost the patient adequate diagnosis and treatment; when negative, antimalarial medication must continue to be withheld. [22]

The sensitivity of the pLDH band was lower than that of the HRP-2 band, possibly due to the tropical climate in Gabon. [23] The high sensitivity of the HRP-2 band in this population suggests that HRP-2 gene deletions are unlikely to be common in our population. However, ongoing surveillance is important to document the emergence of HRP-2 gene deletions, particularly in settings where RDTs are widely used and most devices on the market test for HRP-2 alone. The S-DUO RDT also showed substantial agreement with the SD-Bioline RDT; discrepancies were mainly due to the higher positivity rate of the pLDH component compared to that for SD-Bioline.

The CRP band’s lower sensitivity (86·9%, [95%CI: 80–92%]) to detect 20 mg/L or above of the S-DUO RDT compared to the antigenic component could be anticipated. With any quantitative test, an associated level of variability is expected, often calculated as the coefficient of variation; [24] therefore, we expect to see a certain amount of misclassification around the detection limit of 20 mg/L. In addition, all classification groups in our acutely febrile cohort had a mean CRP value above 20 mg/L. This result indicates that a cut-off value of 20 mg/L is inadequate for distinguishing bacterial from non-bacterial causes of fever in febrile illness in our setting, and CRP band performance requires further optimisation. [25] In addition, the potential economic impact of successful implementation of an optimised CRP band S-DUO RDT, the CRP band estimated at the cost of a dollar more to that of a standalone malaria RDT, is worth pursuing [26].

Our model suggests that CRP can be a specific marker for distinguishing bacterial from non-bacterial infections, albeit as a standalone data point. A mean (SD) value of 41·2 mg/L (40) was able to distinguish bacterial from non-bacterial classification significantly; therefore, we hypothesise that a population-derived cut-off that will improve the sensitivity of 30% and maintain a specificity above 90%, alongside clinical evaluation, in guiding clinical management of acute febrile cases in malaria-endemic regions is feasible. The result of a study in Burkina Faso ascertained the usefulness of a semi-quantitative CRP RDT in reducing antibiotic prescription in the management of acute febrile illness, where 80·5% of children under five had a CRP level > 40 mg/L. Still, only 12·4% had confirmed bacterial infection [27]. A novel electronic algorithm in Tanzania proposed a CRP cut-off of > 80 mg/L [28]. The relationship between CRP and parasite density has also been documented elsewhere in sub-Saharan Africa. It highlights the potential for this combo malaria/CRP RDT to also be utilised to assess the severity of malaria infections when the malaria antigen band is positive. [29, 30] Combination malaria RDT (dual antigen and CRP) developers should take these data in consideration; however, further investigation with a larger sample size in areas where *P. falciparum* is prevalent is required [31].

The 170 participants excluded from the PP population were all enrolled during the first three months of the study. During that time, the S-DUO RDT was performed incorrectly by study staff, resulting in a systematic error and invalid results. The S-DUO and SD-Bioline RDT devices are manipulated differently (assay procedural steps), however staff had performed only the SD-Bioline procedure they were previously experienced with for both devices. This error should not have introduced bias as it was during a defined enrolment period. There were no intrinsic deficiencies with the RDT devices, staff did not encounter any difficulties with the devices for blood transfer, and were quickly accustomed to the S-DUO RDT. The CRP reference test used in this analysis, the NycoCard reader, has a broad but relatively limited measurement range of five to 120 mg/L for serum/plasma, which made further evaluation of CRP levels difficult; it is likely that participants with levels beyond and below this range would otherwise have been identified, which may have impacted our CRP correlation analyses. Antibiotic prescribing practice in our study might not reflect routine practice in Gabon (7% [27/415] of the study population were prescribed antibiotics) [32].

In conclusion, the S-DUO RDT adequately diagnosed malaria in our study population compared to microscopy and SD-Bioline RDT. S-DUO RDT has the potential to complement clinical management of non-malarial febrile illness in malaria-endemic regions if a specific detection limit of the CRP component is determined; if determined, the finding of (1) a positive malaria test and negative CRP result and no clinical signs would provide reassurance that malaria could be treated on an outpatient basis without administration of antibiotics, (2) a negative malaria test and negative CRP result would suggest symptomatic management and non-prescription of an antibiotic. It is also important to note that not all bacterial infections require the prescription of antibiotics, and in some cases, indiscriminate use can be counterproductive to the patient. An optimal cut-off value of CRP based on malaria transmission intensity (moderate transmission in our population, cut off > 40 mg/L), its integration into duo RDT, and the utility of these tests to change clinical practice may be instrumental to reducing the indiscriminate use of antibiotics.

## Data Availability

Data will be made available upon request after the study results are published. Please contact the corresponding author: agnandjis@cermel.org.
