# Correction: Performance evaluation of a combination *Plasmodium* dual-antigen CRP rapid diagnostic test in Lambaréné, Gabon

**DOI:** 10.1007/s15010-024-02415-6

**Published:** 2024-10-09

**Authors:** Ayodele Alabi, Fungai P. Musangomunei, Fabrice Lotola-Mougeni, Juste C. Bie-Ondo, Kristin Murphy, Paulin N. Essone, Anita L. Kabwende, Saidou Mahmoudou, Aurélien Macé, Victoria Harris, Michael Ramharter, Martin P. Grobusch, Maria Yazdanbakhsh, B. Leticia Fernandez-Carballo, Camille Escadafal, Peter G. Kremsner, Sabine Dittrich, Selidji T. Agnandji

**Affiliations:** 1 Biomedicine and Social Sciences Research Group, Department of Biologicals and Therapeutics, Centre de Recherches Médicales de Lambaréné, CERMEL, BP 242, Lambaréné, Gabon; 2 Institut für Tropenmedizin, Universitätsklinikum Tübingen and German Centre for Infectious Diseases Research (DZIF), Tübingen, Germany; 3 Leiden University Center for Infectious Diseases (LU-CID), Leiden University Medical Center, Leiden, The Netherlands; 4 London School of Hygiene and Tropical Medicine, London, UK; 5 FIND, Geneva, Switzerland; 6 Department of Tropical Medicine, Department of Internal Medicine I, Bernhard Nocht Institute for Tropical Medicine, University Medical Centre Hamburg-Eppendorf, Hamburg, Germany; 7 Center of Tropical Medicine and Travel Medicine, Department of Infectious Diseases, Amsterdam Public Health, Amsterdam University Medical Centres, Location Amsterdam, Amsterdam Infection & Immunity, University of Amsterdam, Amsterdam, The Netherlands; 8 Nuffield Department of Medicine, University of Oxford, Oxford, UK; 9 Deggendorf Institut of Technology, European Campus Rottal-Inn, Deggendorf, Germany; 10 Institute of Medical Microbiology, University Hospital Münster, Domagkstraße 10, 48149 Münster, Germany

**Infection**

10.1007/s15010-024-02366-y

Due to complications in figure processing, Fig. 2 of this article was only partially typeset. See below for the incomplete Fig. 2:

**Fig. 2:**
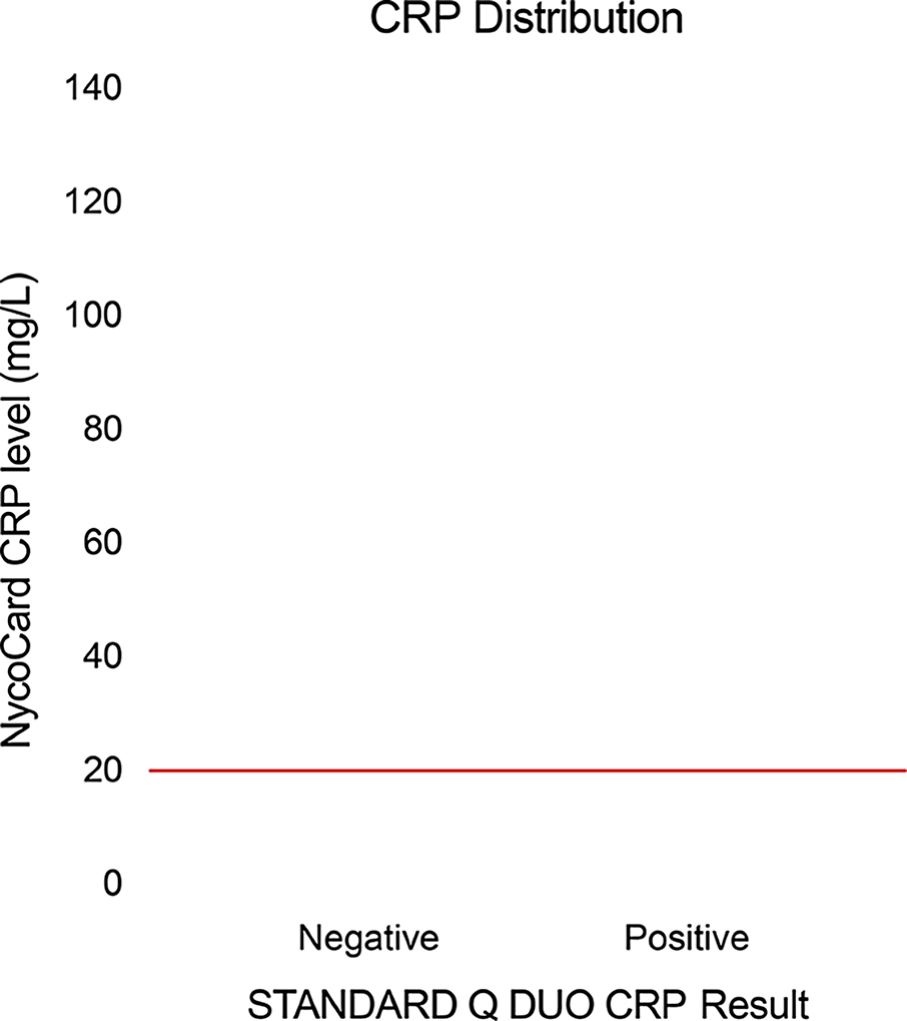
CRP distribution by Nycocard device vs. S-DUO RDT result in the Per Protocol population. Median values and interquartile ranges shown in box plots, the dots represent individual samples. The red line indicates detection limit of S-DUO RDT (20 mg/L)

The correct and complete Figure is as follows:

**Fig. 2:**
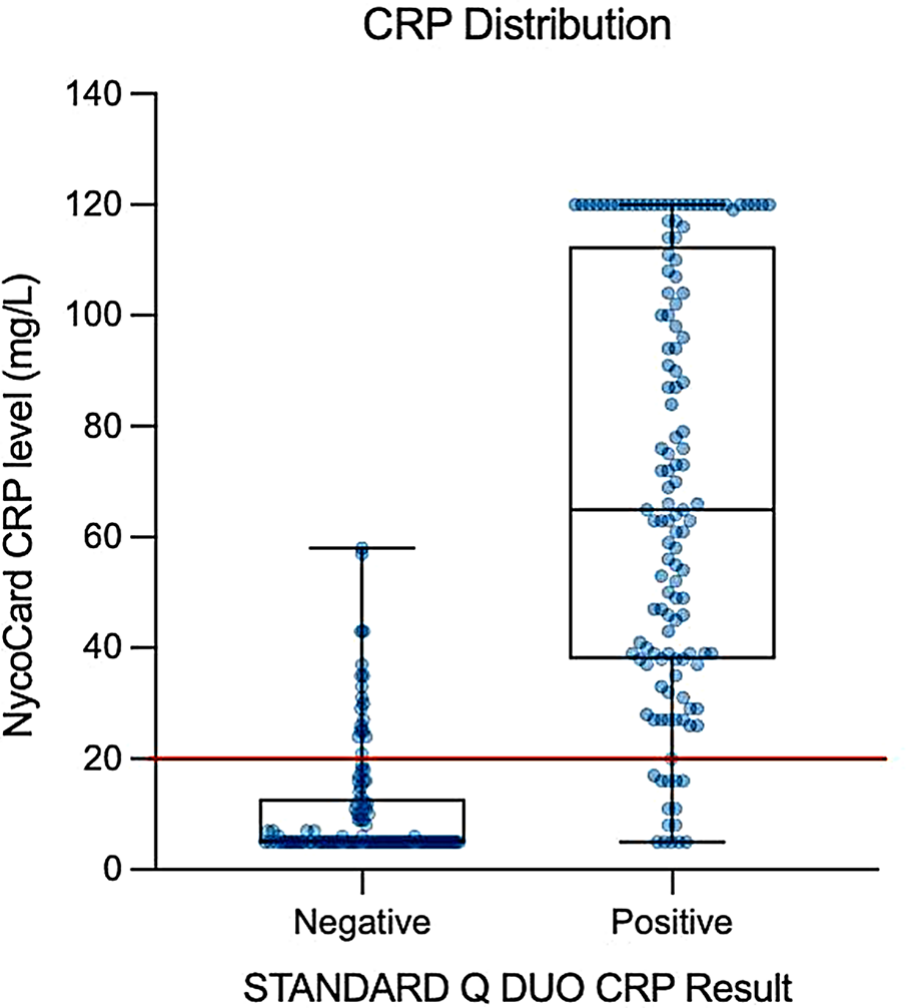
CRP distribution by Nycocard device vs. S-DUO RDT result in the Per Protocol population. Median values and interquartile ranges shown in box plots, the dots represent individual samples. The red line indicates detection limit of S-DUO RDT (20 mg/L)

